# The Neural Correlates of Grasping in Left-Handers: When Handedness Does Not Matter

**DOI:** 10.3389/fnins.2018.00192

**Published:** 2018-04-03

**Authors:** Chiara Begliomini, Luisa Sartori, Maria G. Di Bono, Sanja Budisavljević, Umberto Castiello

**Affiliations:** ^1^Dipartimento di Psicologia Generale, Università degli Studi di Padova, Padua, Italy; ^2^Padua Neuroscience Center, Padua, Italy; ^3^UNICEF Montenegro, Podgorica, Montenegro

**Keywords:** reach-to-grasp, handedness, left-handers, functional magnetic resonance imaging, dynamic causal modeling

## Abstract

Neurophysiological studies showed that in macaques, grasp-related visuomotor transformations are supported by a circuit involving the anterior part of the intraparietal sulcus, the ventral and the dorsal region of the premotor area. In humans, a similar grasp-related circuit has been revealed by means of neuroimaging techniques. However, the majority of “human” studies considered movements performed by right-handers only, leaving open the question of whether the dynamics underlying motor control during grasping is simply reversed in left-handers with respect to right-handers or not. To address this question, a group of left-handed participants has been scanned with functional magnetic resonance imaging while performing a precision grasping task with the left or the right hand. Dynamic causal modeling was used to assess how brain regions of the two hemispheres contribute to grasping execution and whether the intra- and inter-hemispheric connectivity is modulated by the choice of the performing hand. Results showed enhanced inter-hemispheric connectivity between anterior intraparietal and dorsal premotor cortices during grasping execution with the left dominant hand (LDH) (e.g., right hemisphere) compared to the right (e.g., left hemisphere). These findings suggest that that the left hand, although dominant and theoretically more skilled in left handers, might need additional resources in terms of the visuomotor control and on-line monitoring to accomplish a precision grasping movement. The results are discussed in light of theories on the modulation of parieto-frontal networks during the execution of prehensile movements, providing novel evidence supporting the hypothesis of a handedness-independent specialization of the left hemisphere in visuomotor control.

## Introduction

The neural correlates of grasping in humans have been intensively investigated by means of neuroimaging and brain stimulation techniques (for reviews see Castiello, 2005; Castiello and Begliomini, 2008; Filimon, 2010). These studies mainly rely on neurophysiological findings in the attempt to identify in humans a cortical network similar to that described in monkeys, in which the anterior intraparietal area (AIP), the ventral (F5), and the dorsal (F2) premotor cortices play a key role for the execution of grasping movements (Murata et al., 1997; Rizzolatti and Luppino, 2001; Raos et al., 2004; see Castiello, 2005; Castiello and Begliomini, 2008 for reviews). The majority of these studies highlighted that grasping actions performed with one hand or the other are usually mirrored by an asymmetric recruitment of the two hemispheres in functional terms (left hand/right hemisphere vs. right hand/left hemisphere) (Brouwer et al., 2001; Johnson-Frey et al., 2005; Basso et al., 2006; Pollok et al., 2006; Begliomini et al., 2008; Martin et al., 2011; Kourtis et al., 2014). However, in some cases ipsilateral activations within motor-related areas have also been reported (Kim et al., 1993; Volkmann et al., 1998; Baraldi et al., 1999; Kobayashi et al., 2003; Verstynen et al., 2005).

To date, most of these neuroimaging studies have focused on right-handed participants performing grasping movements with their right hand, neglecting a basic feature of the human body and motor behavior: the presence of two functional hands, physically symmetrical but functionally distinct. It has been estimated that 90% of humans show the tendency to use their right hand for interacting with objects and the environment, while the left hand plays a supporting role. However, the remaining 10% of the population shows the opposite functional pattern with the left hand as a dominant one (Perelle and Ehrman, 1994). Whether the mechanisms underlying the motor control of the left-handers simply mirror that of the right-handers has been the focus of behavioral studies. In general, these studies simply observe whether there is a tendency, in both right- and left-handers, to choose a particular hand to perform a given motor task, such as grasping (Gonzalez et al., 2006, 2007; Gonzalez and Goodale, 2009; Stone et al., 2013; Main and Carey, 2014; Stone and Gonzalez, 2015). Overall, these studies indicate the left hemisphere/right hand ensemble as specialized for grasping, independently from handedness, and the right hemisphere/left hand ensemble as critical in haptic tasks (Stone et al., 2013; Stone and Gonzalez, 2015). What is less well understood is how the human brain controls grasping movements with the right or the left hand, in both right- and left-handers, as there are only a few imaging studies focusing on this issue. An unpublished report (Culham et al., unpublished) considered right-handers performing grasping movements with either the right or the left hand toward 3D targets while being scanned. These results indicated that grasping with either hand recruits AIP bilaterally, with a significantly stronger and more extended recruitment of the hemisphere contralateral to the hand used. Similar evidence has been provided also by Martin et al. (2011) and very recently also by Tzourio-Mazoyer et al. (2015): both studies show that while right-handers are characterized by a clear asymmetric pattern of brain activity (left hemisphere/right hand; right hemisphere/left hand), left-handers show a bilateral recruitment of brain regions involved in motor control, independently of the hand used. In another study (Begliomini et al., 2008) right- and left-handers were scanned while performing a precision grip task with the right or the left hand. Results confirmed the crucial role of the bilateral AIP: this region, together with the right dorsal premotor cortex (dPMC) and the right cerebellum appeared to be significantly modulated by hand and handedness, in both right- and left-handers. The fact that both AIPs and the cerebellum showed a similar pattern of modulation according to the hand and handedness provided support to the existence of a cerebellum-AIP connections in humans, as already described in monkeys (Clower et al., 2005). Effective connectivity approach (Dynamical Causal Modeling—DCM; Friston et al., 2003) was recently adopted to further test the idea that in right-handers the contribution of the two hemispheres to the execution of grasping movements might vary according to the performing hand (Begliomini et al., 2015). The results highlighted strengthened inter-hemispheric connections between dPMCs during grasping with the left non-dominant hand and further emphasized the fundamental contribution of the dPMC in monitoring the fingers' configuration, suggesting that when the less skilled hand is used, additional control is required.

**Figure 1:**
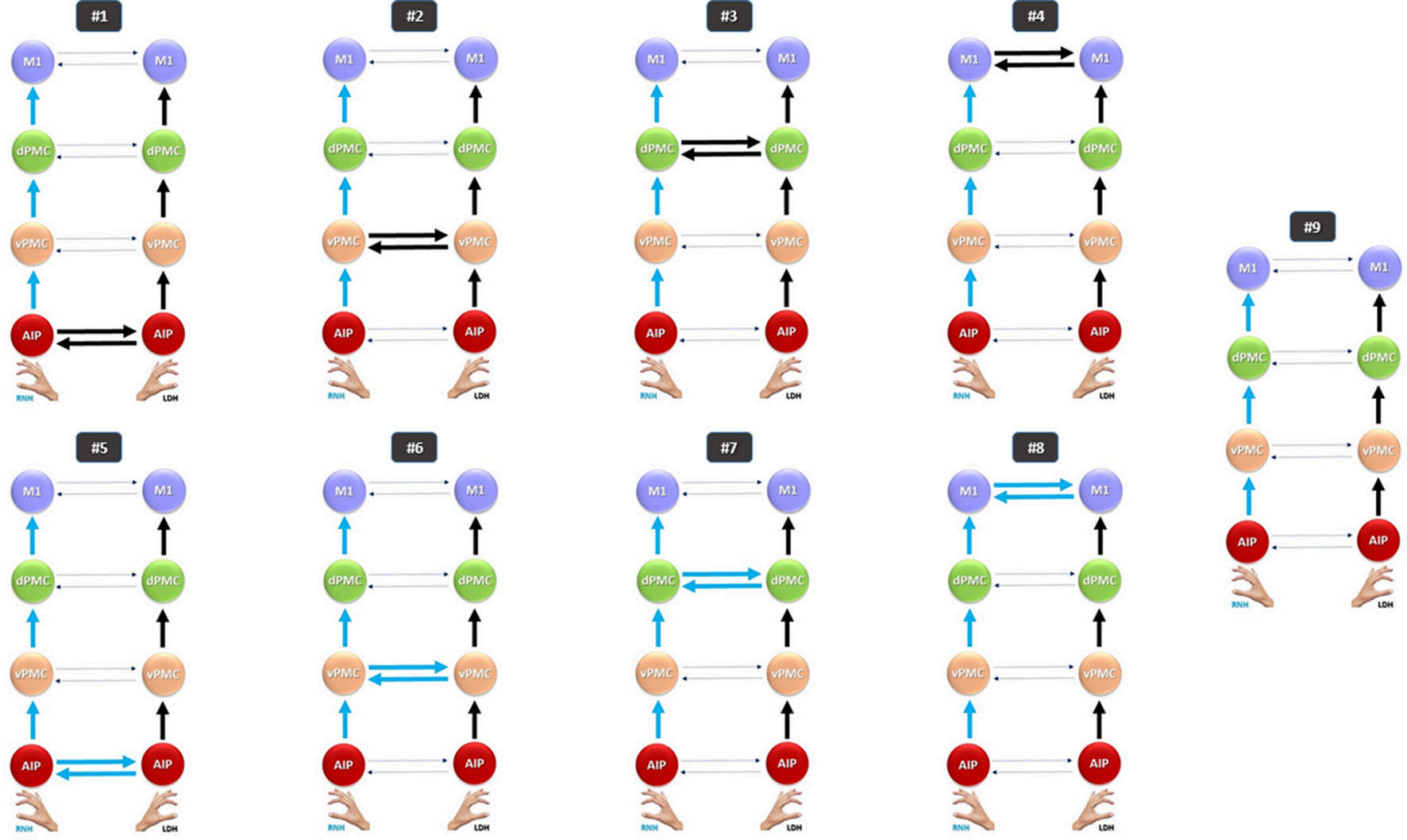
Models tested for the RFX Bayesian Model Selection (BMS). Models #1–4 belong to the LDH family; models #5–8 refer to the RNH family; model #9 (null model) does not belong to any “family” and does not include any inter-hemispheric connection. Solid blue arrows indicate connections (both intra- and inter-hemispheric) modulated by Precision Grip movements performed with the RNH; solid black arrows indicate connections modulated by PG movements performed with the LDH; dotted arrows indicate connections not affected by modulation effects. AIP, Anterior Intraparietal; vPMC, Ventral Premotor Cortex; dPMC, Dorsal Premotor Cortex; M1, Primary Motor Cortex; LH, Left Hemisphere; RH, Right Hemisphere; RNH, Right Non Dominant Hand; LDH, Left Dominant Hand.

For the first time here we explore the contribution of both hemispheres to the execution of a precision grasping task, performed by left-handers with the left or the right hand. Specifically, we aim to observe (i) whether the execution of a precise grasp involves the grasping network according to a specular schema, so that grasping with the left dominant hand (LDH) mainly recruits the right hemisphere, whereas grasping with the right non-dominant hand (RNH) mainly recruits the left hemisphere; and (ii) whether left hand dominance influences intra-hemispheric connectivity patterns among areas belonging to the grasping circuit, as observed in a previous study in right handers (Begliomini et al., 2015). Relying on structural and functional evidence obtained in both humans and monkeys (see Table 1) (iii) we also investigated whether inter-hemispheric effective connectivity between homologous areas could be affected either by the use of the right hand, which is non-dominant in left-handers, or rather by the use of the left hand, which is supposed to be dominant, but potentially less-skilled. We considered the four key regions of the “grasping network,” namely the AIP, the ventral premotor cortex—vPMC, dPMC and the primary motor cortex—M1 (Castiello and Begliomini, 2008), hypothesizing that connections between homologous areas of the two hemispheres would be modulated during precision grasping task, according to the performing hand. In this respect, three possible scenarios were considered (Figure 1):

1. The execution of precision grasping with the LDH modulates contralateral intra-hemispheric *and* inter-hemispheric connections between homologs areas (models #1–4);
2. The execution of precision grasping with the RNH modulates contralateral intra-hemispheric *and* inter-hemispheric connections between homologs areas (models #5–8);
3. The execution of precision grasping with either the LDH or the RNH modulates contralateral intra-hemispheric but *not* inter-hemispheric connections between homologs areas (model #9).

**Table 1:** Studies demonstrating the existence of inter-hemispheric connections between grasping areas considered in the present study.

**Connection**	**Non-human primate studies**	**Human primate studies**
*AIP—AIP*		Culham and Valyear, 2006; Begliomini et al., 2008; Le et al., 2014; Tunik et al., 2005; Rice et al., 2006; Davare et al., 2007
*vPMC—vPMC*	Boussaoud, 1995; Dancause et al., 2007	
*dPMC—dPMC*	Marconi et al., 2003	Begliomini et al., 2008, 2015
*M1—M1*	Jenny, 1979; Leichnetz, 1986; Rouiller et al., 1994	Davare et al., 2007

*AIP, Anterior IntraParietal; vPMC, ventral PreMotor Cortex; dPMC, dorsal PreMotor Cortex; M1, Primary Motor Cortex*.

## Methods

### Participants

Sixteen participants (11 females; age range: 21–32 years; mean age: 26.1 years) participated in the experiment. All participants had normal vision and had no history of neurological, psychiatric or motor disease. Left hand dominance was evaluated by means of the Edinburgh Handedness Inventory (Oldfield, 1971), and only participants with a laterality score index ranging from 0.6 to 1 (strongly left-handed) were included. Before undergoing the fMRI session all participants underwent a safety screening and received all relevant information about the experimental procedure and data treatment. The study was carried out according to the guidelines of the Ethics Committee for Clinical Practice of Padova University Hospital. All participants gave written informed consent in accordance with the Declaration of Helsinki. The study has been supported by a grand awarded from the Italian Ministry for Education, University and Research to Chiara Begliomini (CPDA117759/11).

### Experimental stimulus

The stimulus was a spherical MR-compatible object of 3 cm diameter, presented at a distance allowing the comfortable execution of a grasping movement, and which was the same for both hands. A regular geometric shape was chosen to allow for comparisons with previous neurophysiological (Gallese et al., 1994; Umilta et al., 2007) and neuroimaging (e.g., Begliomini et al., 2007a) studies. Stimulus dimension was selected in order to elicit a precision grip, that is the opposition of thumb and index finger. This kind of prehensile action has been well described in humans at both neural (Ehrsson et al., 2001; Frey et al., 2005; Culham and Valyear, 2006; Begliomini et al., 2007b, 2014; Turella and Lingnau, 2014) and behavioral level (e.g., Jeannerod, 1981, 1984; Castiello et al., 1993; Savelsbergh et al., 1996; Cuijpers et al., 2004; see Smeets and Brenner, 1999 for a review). In addition, neuroimaging studies have highlighted how planning and execution of precision grip movements are characterized by a larger involvement of the fronto-parietal network with respect to other types of grasping (e.g., whole hand grasp – Begliomini et al., 2007a,b; see Filimon, 2010 for a review).

### Experimental setup

**Figure 2:**
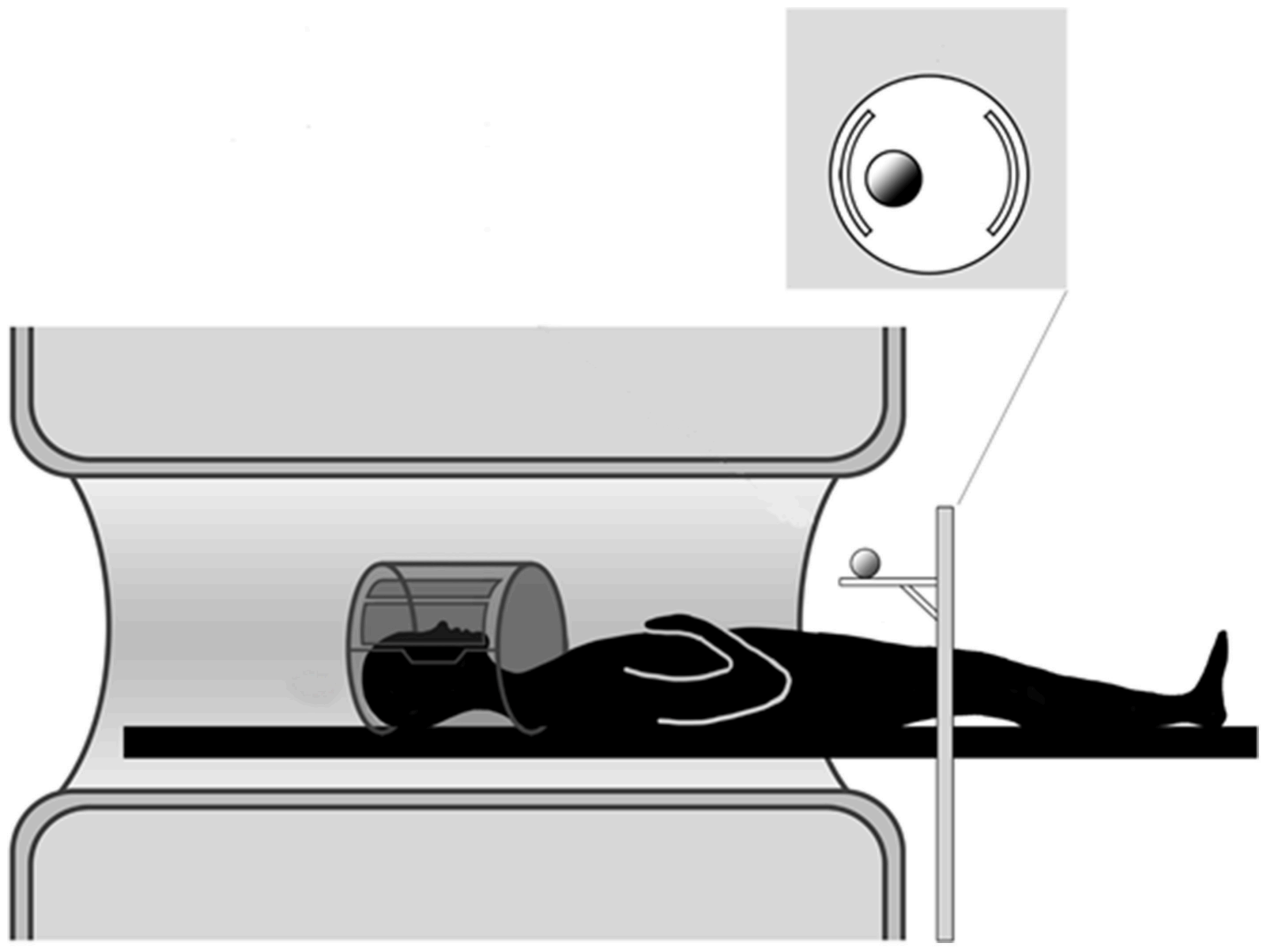
Experimental setup. The participant is lying in the MR scanner bore and the platform allows the presentation of a stimulus at a comfortable distance. A pillow, slightly tilting the head, allows for a direct viewing of the stimulus.

The stimulus was presented on a small circular MR compatible table (Figure 2). Participants' upper arms were kept still and tight to the body with an elastic band as to minimize possible head motion induced by arm movements. In order to ensure a consistent starting position for both hands and comparable for both hands, all participants wore a plastic belt with a pad in the middle (e.g., on the body midline). They were instructed to keep both hands placed on the pad in a relaxed position with the palms facing down between trials. The participants' head was supported by a foam pillow, in order to have a ~30° tilted position, to allow for a direct view of the stimuli without mirrors (Culham et al., 2003; Cavina-Pratesi et al., 2007).

### Task procedures

The participants were instructed to perform a grasping movement toward the stimulus at a natural speed, without any time restraint, using a precision grip with either the LDH or the RNH hand according to a sound delivered by means of pneumatic MR-compatible headphones (right hand: low pitch - duration: 200 ms; frequency: 1,7 kHz; left hand: high pitch - duration: 200 ms; frequency: 210 Hz). Although the stimulus was constantly visible, participants were instructed not to begin the movement until after hearing the sound. An operator monitored the entire experiment from the control room, checking that the task was performed correctly. Participants were explicitly instructed to look at the object throughout action execution.

### Experimental design

The experiment adopted a mixed event-related design, with performing hand (LDH, RNH) manipulated as within-subjects factor (within runs). Trials involving the same hand were gathered in sequences varying from four to eight elements, as to minimize task-switching related brain activity, induced by frequent changes of the effector (Culham et al., 2003). Inter-stimulus interval (ISI) was randomized across trials, varying from 3 to 8 s according to a “long exponential” probability distribution (Hagberg et al., 2001). The whole experiment consisted of 120 trials (60 per hand), divided into 2 runs of 60 trials each.

### Imaging parameters

Images were acquired by means of a 1.5 Tesla scanner (Siemens Avanto) with a standard 8-channels coil. Functional images measuring the blood oxygenation level-dependent (BOLD) contrast were acquired with a gradient-echo, echo-planar (EPI) T2^*^-weighted sequence covering the whole brain volume (37 continuous axial slices, descending order, 56 × 64 voxels, 3 mm × 3 mm × 3.3 mm resolution, FOV = 196 mm × 224 mm, flip angle = 90°, TE = 49 ms). 114 volumes were acquired for each of the two runs (5 min and 42 s for each run, for a total acquisition time of 11 min and 24 s). A high-resolution structural T1-weighted image was acquired for each participant (3DMP-RAGE, 176 axial slices, 1 mm isotropic voxel, no interslice gap, data matrix 256 × 256, TR = 1,900 ms, TE = 2.91 ms, flip angle = 15°).

### Data analysis

#### Data preprocessing

Functional data underwent spatial pre-processing and analysis with the SPM (Statistical Parametric Mapping, www.fil.ion.ucl.ac.uk/spm), version 12. The first four scans of each functional run were excluded from data analysis to allow for T1 equilibrium state. For each participant, the time series were temporally realigned to the middle slice and were corrected for head motion (translations/rotations), taking the first volume of the series as a reference. The structural image was then co-registered to the mean of all functional images previously corrected for signal intensity inhomogeneities. Functional images were then normalized according to the MNI152 template (Montreal Neurological Institute, http://www.mni.mcgill.ca) implemented in the software SPM12, and were finally smoothed using a 6 × 6 × 6.6 mm FWHM 3D Gaussian kernel (twice the native voxel size).

#### General linear model

At the first level, for each participant, movements performed either with the LDH or the RNH were modeled as single events with an assumed duration of about 1.5 s on the basis of behavioral observations preceding the experimental session (this was done to allow the participants become familiar with the experimental setup). Trials timing was defined on the basis of the onset of the cueing sound indicating the hand to be used to perform the grasping action. Movements performed with either the LDH or the RNH were modeled as separate regressors, and were convolved with a canonical, synthetic HRF (haemodynamic response function) to produce individual models (Henson, 2001). A General Linear Model (Holmes et al., 1997) was run for each single subject, including the two regressors of interest (LDH; RNH) plus additional regressors of no interests (head motion parameters created during the realignment stage; trials for which the participants did not react/did not perform the movement correctly). The functional time series were concatenated over the two sessions, and two additional regressors of no interest were added to the model to account for possible session effects.

#### DCM models

The aim of DCM (Friston et al., 2003; Stephan et al., 2007) is to identifying possible causal relationship among brain regions through the comparison of several different causality hypotheses (e.g., models) involving a given pool of a priori identified brain regions. In the present study, this approach was adopted to characterize how the two hemispheres of a group of left-handed participants contribute to the execution of a precision grasping movement performed with the LDH or the RNH. Effective connectivity between areas belonging to the grasping circuit in humans was explored, hypothesizing nine different scenarios (Figure 1). The considered regions are: AIP, vPMC, dPMC, and M1 (Castiello, 2005; Castiello and Begliomini, 2008; Filimon, 2010). Here the basic idea eas that the performing hand (LDH or RNH) could modulate causal connections between homologous areas of the two hemispheres (e.g., right M1-left M1) according to the models described in Figure 1. First, *intra-hemispheric* connections among the grasping key regions (AIP, vPMC, dPMC, and M1) were considered, according to the results obtained by single cell recordings performed on macaques (see Table 1) and relying on the model described by Castiello and Begliomini (2008). This first step was performed to confirm the involvement of the right hemisphere in coding for grasping performed with the LDH and the left hemisphere in coding for grasping performed with the RNH as a starting point. Second, *inter-hemispheric* connections between homologous areas in the two hemispheres were explored. Concerning this step, it has to be emphasized that previous neurophysiological data represented the main reference point for connections between dPMC, vPMC, and M1. For AIP we mainly relied on the neuroimaging (Culham et al., 2006; Begliomini et al., 2008, 2015) and neurostimulation results (Tunik et al., 2005; Rice et al., 2006; Le et al., 2014) previously reported in humans. Overall these studies agree that a bilateral recruitment of the AIP is crucial for grasping execution. For all participants nine different models were considered, assuming nine different connectivity hypotheses (see Figure 1). Anatomical *context-independent* models (DCM-A matrix) were formulated on the basis of the abovementioned literature and the performing hand was adopted as a *context-dependent* modulatory agent on the forward connections (LDH; RNH—DCM-B matrix). AIP was included as a driving input (matrix C) for both hemispheres since the visuomotor analysis of the object target of the action represents an essential requirement for the successful accomplishment of a grasping action. In this sense, both neurophysiological and neuroimaging support the consideration of AIP as a crucial region (Binkofski et al., 1998, 1999; Castiello, 2005; Frey et al., 2005; Rice et al., 2006, 2007; Begliomini et al., 2007b; Castiello and Begliomini, 2008): for this reason. Any possible hypothesis related to stimulus-response coupling mechanism (sound → performing hand) was not taken into account, since the present study was focused on grasping execution, rather than previous stages such as planning. According to the model (envisaged by Castiello and Begliomini, 2008), the modulation induced by the act of performing a precision grasp is supposed to spread through ipsilateral connections from AIP to vPMC, and from vPMC to dPMC. The following connection is supposed to link dPMC with M1, which is assumed to be the last step of the considered models (see Figure 1). Models #1–4 were considered as belonging to the “LDH” family, given their assumption of inter-hemispheric interactions between homologous grasping areas as modulated by precision grip movements performed with the LDH (model #1: right AIP ↔ left AIP; model #2: right vPMC ↔ left vPMC; model #3: right dPMC ↔ left dPMC; model #4: right M1 ↔ left M1). Models #5–8 hypothesize the same architecture, but assume that inter-hemispherical connections between homologous areas are influenced by precision grip movements performed with the RNH (“RNH” family; model #5: left AIP ↔ right AIP; model #6: left vPMC ↔ right vPMC; model #7: left dPMC ↔ right dPMC; model #8: left M1 ↔ right M1). The “null” model hypothesized no inter-hemispheric connection between the two hemispheres (#9), to test the possibility that the hemispheres do not interact with each other while performing grasping movements with either the LDH or the RNH.

#### VOI definition

For each region included in the nine models the relevant time series was obtained from the fMRI data of each individual participant from the General Linear Model performed at the first level. The selection of VOIs was performed on both anatomical and functional bases: (i) for all participants, the average effect of the experimental manipulation (precision grip movements performed with LDH + precision grip movements performed with RNH; *p* < 0.001, uncorrected for multiple comparisons) was tested by means of a t-contrast, in order to detect brain activity underlying both movements; (ii) a Small Volume Correction (Worsley et al., 1996) was conducted on the resulting activation by considering the cytoarchitectonic probabilistic maps provided by the toolbox Anatomy (Eickhoff et al., 2007) as searching areas. The following maps were considered: AIP (Choi et al., 2006; Scheperjans et al., 2008), vPMC (Amunts et al., 1999), dPMC (Genon et al., 2016, 2018), and M1 (Geyer et al., 1996). Then, the first set of coordinates observed for each area (AIP left, AIP right, vPMC left, vPMC right, dPMC left, dPMC right, M1 left, and M1 right) was selected for the creation of the VOI. Concerning M1, the “hand knob” (Yousry et al., 1997) was adopted as the anatomical landmark to identify the set of coordinates for the creation of the VOI. For all participants, a spherical VOI of 5 mm radius was considered, built around the most significant set of coordinates detected through the SVC. This procedure was performed for each of the 8 regions included in the analysis. The time series extraction considered the “effects of interest” (t-contrast) adjusted for a F-contrast testing for the “effects of interest” and excluding any other regressor of no interest (motion parameters, errors, missed trials). The percentage of variance observed for each region was above 80% in all cases, and all VOIs included at least 10 voxels.

#### Model estimation and selection

Bayesian Inference (Penny et al., 2004) was performed to verify hypotheses concerning the “origin” of the hypothesized recruitment of ipsilateral regions during precision grip movements performed with the RNH and the LDH. We first verified whether and how (e.g., by means of LDH or RNH) the act of performing a precision grasping movement engages contra- and more importantly ipsilateral grasping regions. Bayesian Model Selection (BMS) was performed by means of random effects analysis (RFX; Stephan et al., 2009; Penny et al., 2010) accounting for the possibility that individual variance can be best described by different models. Model comparison was performed following a two-steps approach: (i) inference at a “family” level (i.e., subsets of models sharing specific peculiarities). In this study, two different families were built, on the basis of the origin of the modulation of inter-hemispheric connections (e.g., LDH-driven models; RNH-driven models). Then, (ii) Bayesian comparison was performed within the “winning” family, in order to reveal the model/s best fitting the data. Also the “null” model was included at this stage of the analysis, as to better explore dynamic causality hypotheses involving the two hemispheres.

## Results

### GLM group analysis results

**Figure 3:**
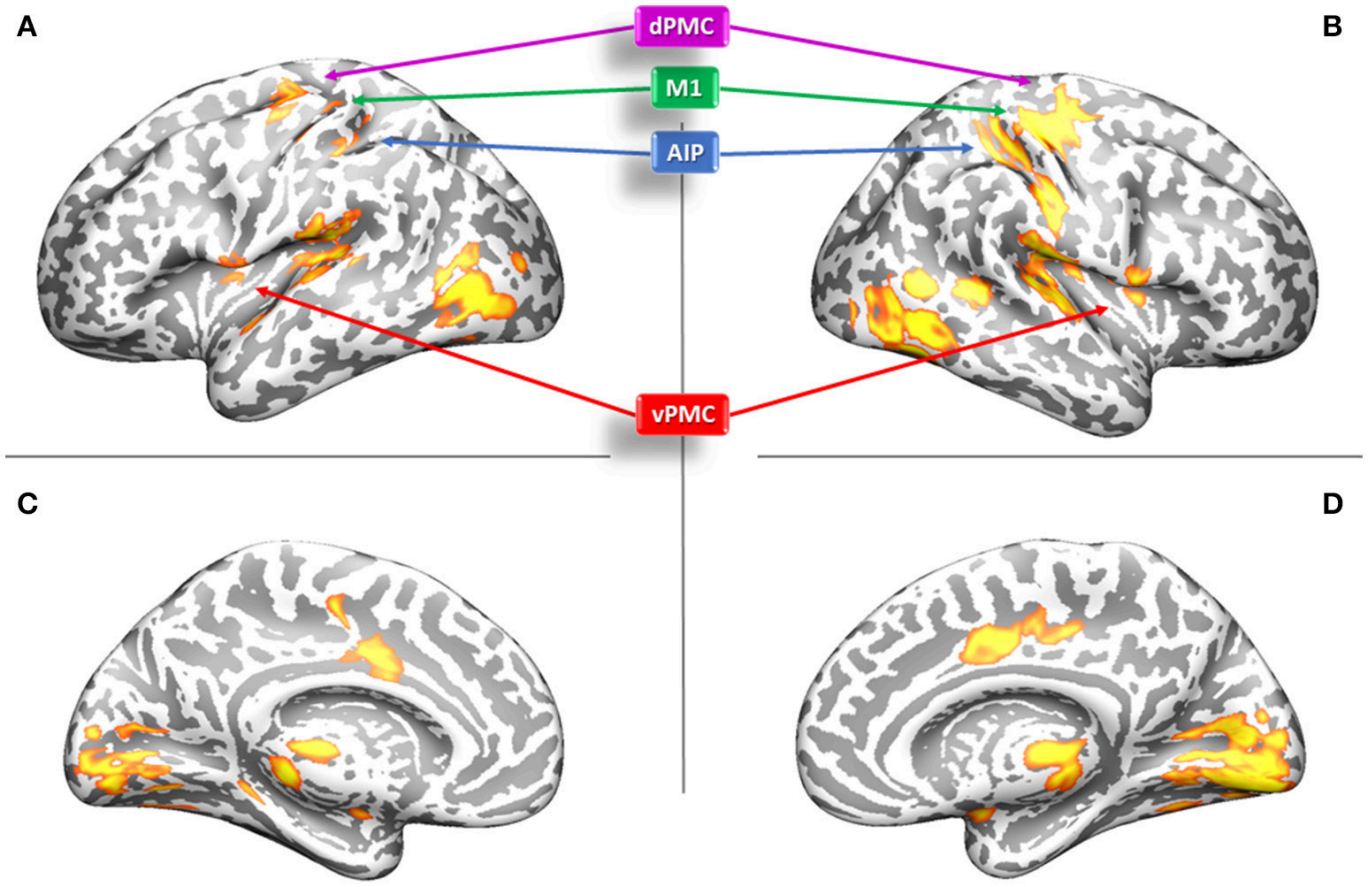
Results of the group analysis (RFX) for the t-contrast RNH+LDH performed on the whole brain. **(A–C)** Left hemisphere, lateral and medial views; **(B–D)**: Right hemisphere, lateral and medial views. AIP, Anterior Intraparietal; vPMC, Ventral Premotor Cortex; dPMC, Dorsal Premotor Cortex; M1, Primary Motor Cortex.

A RFX analysis was conducted (*p* < 0.05, FWE-corrected for multiple comparisons, k ≥ 10) as to verify the involvement of the considered brain regions (AIP, vPMC, dPMC, and M1) in our task. A t-contrast testing for selective effects of precision grip movements performed with the LDH or with the RNH was run within a mask involving the considered brain regions belonging to the grasping circuit. The contrast identified activation in all of these regions, in both hemispheres (see Table 2 and Figure 3).

**Table 2:** Results of the RFX analysis (*p* < 0.05, FWE-corrected for multiple comparisons, *k* ≥ 10).

**CLUSTER level**			**PEAK level**				**MNI**					
***p*(FWE)**	***k***	***p*(unc)**	***p*(FDR)**	***T***	**Z-score**	***p*(unc)**	***X***	***Y***	***Z***	**SIDE**	**REGION**	**BA**
**<0.0001**	**229**	**<0.0001**	**<0.0001**	**10.39**	**6.72**	**<0.0001**	**38**	−**16**	**65**	**RIGHT**	**PRECG**	**4**
			<0.0001	8.36	5.96	<0.0001	52	**–**10	35	**RIGHT**	PRECG	4
			<0.0001	6.66	5.18	<0.0001	35	**–**49	49	**RIGHT**	IPL	40
			<0.0001	6.65	5.12	<0.0001	42	6	59	**RIGHT**	MFG	6
**<0.0001**	**55**	**<0.0001**	**<0.0001**	**7.78**	**5.71**	**<0.0001**	−**38**	−**16**	**62**	**LEFT**	**PRECG**	**4**
			<0.0001	7.26	5.47	<0.0001	**–**31	**–**20	68	LEFT	MFG	6
			<0.0001	5.67	4.64	<0.0001	**–**28	**–**16	59	LEFT	MFG	6
**<0.0001**	**41**	**<0.0001**	**<0.0001**	**7.08**	**5.39**	**<0.0001**	**55**	**10**	**5**	**RIGHT**	**IFG**	**45**
**0.030**	**20**	**<0.0001**	**<0.0001**	**6.77**	**5.23**	**<0.0001**	−**51**	**7**	**15**	**LEFT**	**IFG**	**45**
**0.016**	**18**	**0.0001**	**<0.0001**	**6.50**	**5.10**	**<0.0001**	−**38**	−**30**	**38**	**LEFT**	**IPL**	**40**

*The contrast of interest is precision grip_LDH + precision grip_RNH. PRECG, Precentral Gyrus; IPL, Inferior Parietal Lobule; MFG, Middle Frontal Gyrus; IFG, Inferior Frontal Gyrus. Bolded font indicates the first activation peak of the cluster (in terms of t and Z score)*.

### DCM results

Effective connectivity patterns occurring among the considered brain regions were explored by means of DCM12, provided by the SPM12 toolbox (Wellcome Department of Imaging Neuroscience, London, UK), running in Matlab environment (R2017b, The MathWorks, Natick, MA, USA).

#### Family-wise results

**Figure 4:**
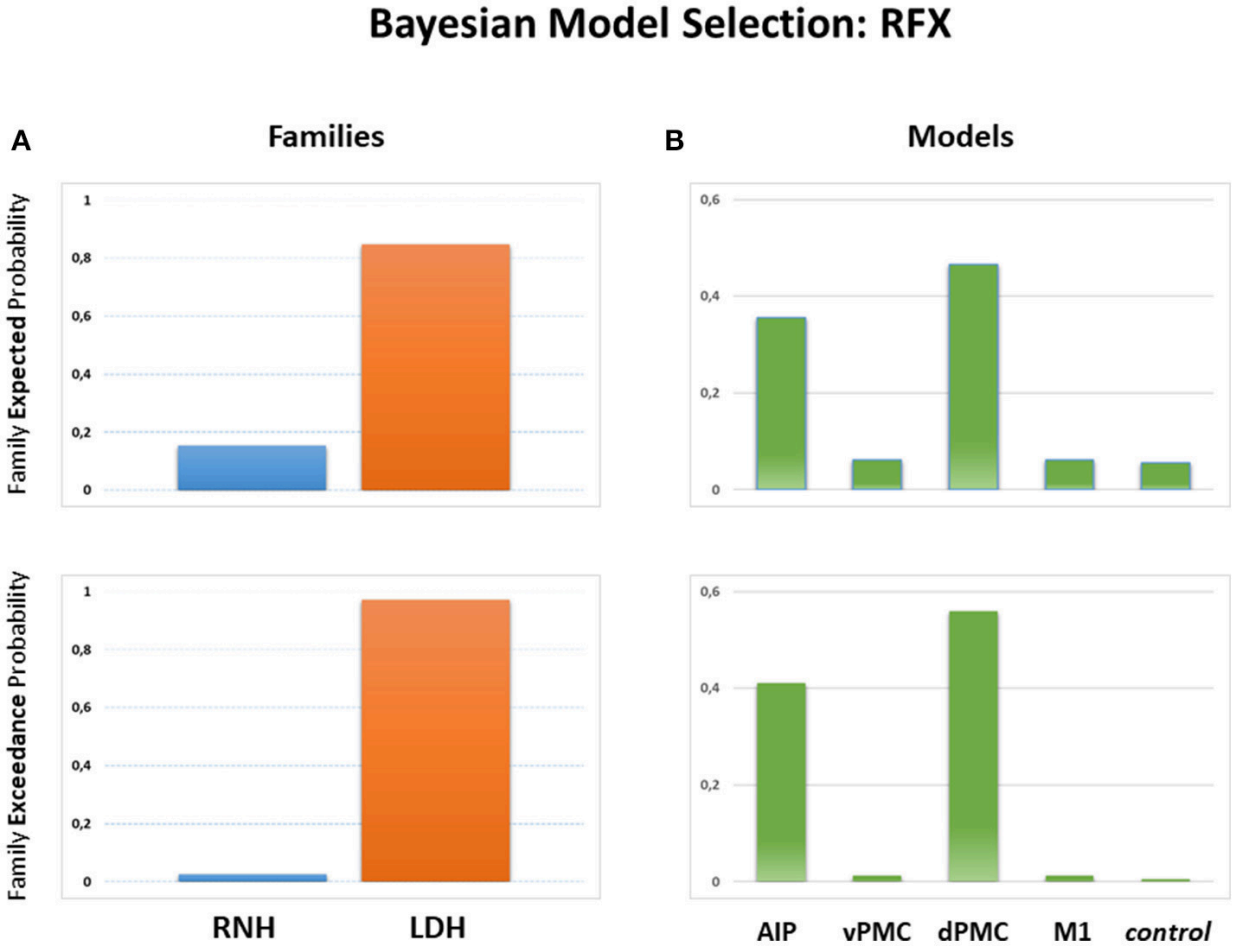
Results of the BMS RFX performed at the family level **(A)** and at the model level **(B)** For both levels, expected (upper panels) and exceedance probabilities (lower panels) are indicated. LDH, Right Dominant Hand; RNH, Left Non-dominant Hand; AIP, Anterior Intraparietal; vPMC, Ventral Premotor Cortex; dPMC, Dorsal Premotor Cortex; M1, Primary Motor Cortex; Control, control model.

BMS was adopted to evaluate which family model (LDH or RNH) better explained the data. The results indicated that the “LDH” family (e.g., movements performed with the LDH—models #1–4) was distinguished by the highest exceedance probability value (0.9732), while the “RNH” family (models #5–8) was consequently associated with a much lower value (0.0268—see Figure 4A). The winning family, LDH, is made up of 4 models sharing the hypothesis of inter-hemispheric connections between homologs areas (AIP, vPMC, dPMC, and M1) as driven by precision grasping executed with the LDH. These models assume this modulation as originating in the right hemisphere and spreading to the left hemisphere through one or more of the considered inter-hemispheric connections.

#### Model-wise results

**Figure 5:**
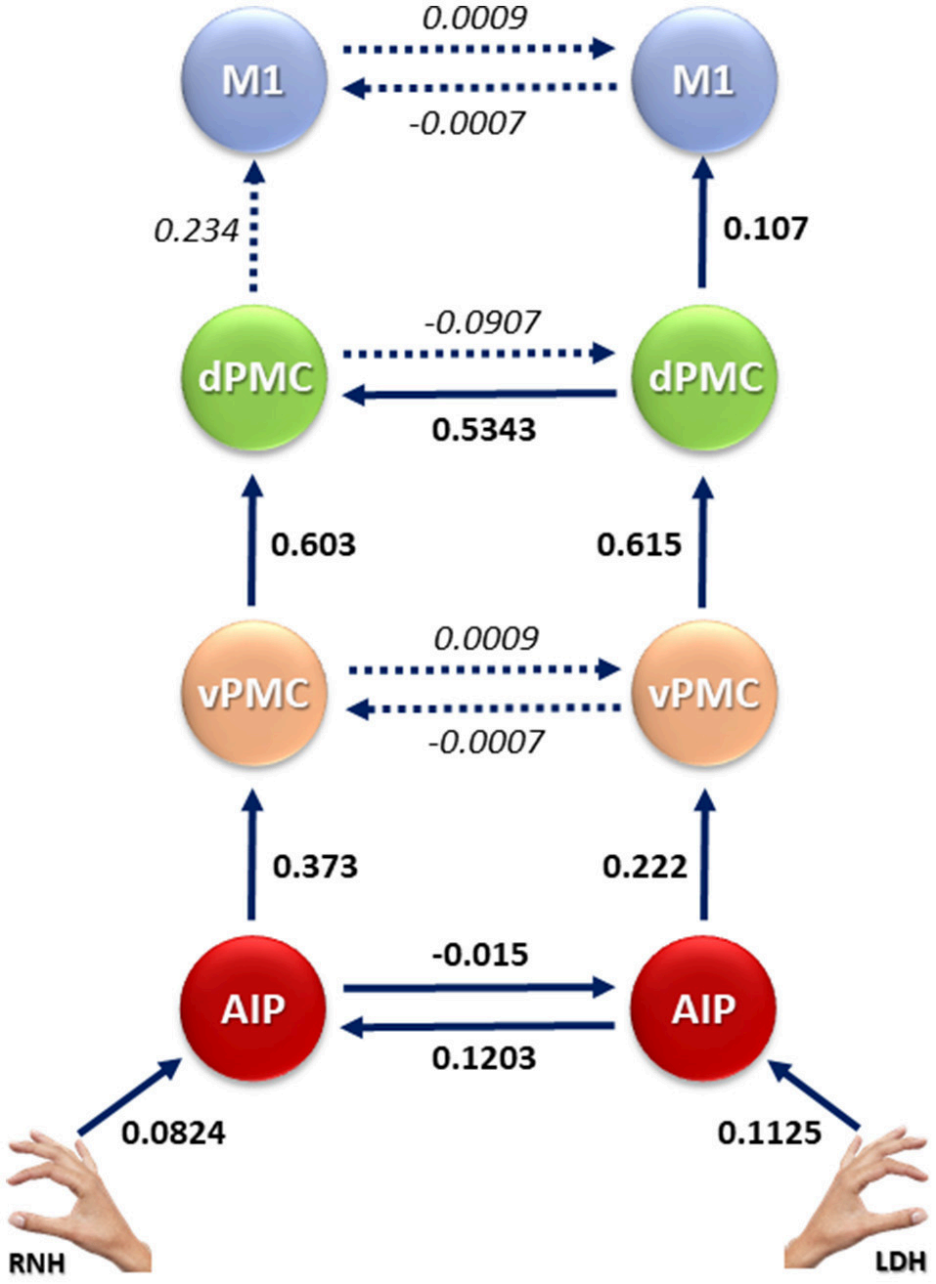
Connection strengths of the tested models. Solid lines indicate significant modulation effects. Group-level averages of the MAP estimates and 95% confidence intervals are illustrated. The mean values were tested against 0. AIP, Anterior Intraparietal; vPMC, Ventral Premotor Cortex; dPMC, Dorsal Premotor Cortex; M1, Primary Motor Cortex.

As a second step, effective connectivity patterns were explored within the “LDH” family, in order to assess which model/s better fits the data. Results show (Figure 4B) that the “dPMC” model is associated with the highest exceedance probability (0.5671), followed by the “AIP” model (0.4113), the “M1” model (0.0123), the “vPMC” model (0.016), and the “null” model (0.0065). These results indicate that, among the considered models, those hypothesizing bidirectional inter-hemispheric modulations occurring either at the AIP (model #1) or at the dPMC (model #3) levels seem to better fit the data. The absence of modulation between hemispheres (model #9) appears to be the most unlikely hypothesis among the considered ones. In order to further characterize the results, parameter estimates of intra-hemispheric connections (DCM-A matrix) resulting from Bayesian Model Averaging (BMA) were extracted and tested against 0 (one-sample *t*-test, *p* < 0.05). This procedure was used to characterize both intra- and inter-hemispheric connection strengths between brain regions involved during the execution of PG movements with the RNH (left hemisphere) or the LDH (right hemisphere). The results are reported in Table 3 and depicted in Figure 5. The statistical analysis showed that grasping with LDH and RNH significantly influences the selected input regions: the left AIP for precision grip movements performed with RNH *t*_(15)_ = 3.29 *p* = 0.004, and the right AIP for precision grip movements performed with the LDH, *t*_(15)_ = 3.87 *p* = 0.001. Concerning the left hemisphere, which is assumed to be primarily recruited when performing precision grip movements with the RNH (Figure 5), two out of three connections between nodes appeared to be significantly modulated [AIP-vPMC: *t*_(15)_ = 8.59, *p* < 0.0001; vPMC-dPMC: *t*_(15)_ = 11.05, *p* < 0.0001]. The connection dPMC-M1 showed a weak trend to significance [*t*_(15)_ = 2.02, *p* = 0.06]. Concerning the right hemisphere, primarily recruited in the control of precision grip movements performed with the LDH (Figure 5), all the connections appeared to be significantly modulated [namely AIP-vPMC: *t*_(15)_ = 5.49, *p* < 0.0001; vPMC-dPMC: *t*_(15)_ = 10.91, *p* < 0.0001; dPMC-M1: *t*_(15)_ = 4.42, *p* = 0.0004]. With regard to inter-hemispheric connections between homologous areas of the two hemispheres (Table 3, Figure 5), the functional link between AIPs appears to be significantly modulated in both directions [L → R *t*_(15)_ = 4.098, *p* = 0.0009; R → L *t*_(15)_ = 4.492, *p* = 0.0004]. While connections between vPMCs did not show any significant modulation effect in either directions, dPMCs connections appears to be significantly modulated in both directions [L → R *t*_(15)_ = 2.069, *p* = 0.0164; R → L *t*_(15)_ = 14.18, *p* < 0.0001]. Differently, connections between M1s did not highlight any significant result [L → R *t*_(15)_ = 0.01, *p* = 0.9981; R → L *t*_(15)_ = 0.01, *p* = 0.9982].

**Table 3:** Results of one-sample *t*-tests performed on the parameter estimates related to input effects, intra-and inter-hemispheric connections within the winning family RNH (*p* < 0.05).

	**INPUT**	**AIP** **LEFT**	**AIP** **RIGHT**	**vPMC** **LEFT**	**vPMC** **RIGHT**	**dPMC** **LEFT**	**dPMC** **RIGHT**	**M1** **LEFT**	**M1** **RIGHT**
AIP LEFT	***t*_(15)_: 3.29** ***p* = 0.0048**		***t*_(15)_: 4.49** ***p* = 0.0004**						
AIP RIGHT	***t*_(15)_: 3.87** ***p* = 0.0014**	***t*_(15)_: −4.09** ***p =* 0.0009**							
vPMC LEFT		***t*_(15)_: 8.59** ***p* <0.0001**			*t_(15)_:* **–***0.05* *p = 0.9614*				
vPMC RIGHT			***t*_(15)_: 5.49** ***p* <0.0001**	*t_(15)_: 0.06* *p = 0.949*					
dPMC LEFT				***t*_(15)_: 11.05** ***p* <0.0001**			***t*_(15)_: 14.18** ***p* <0.0001**		
dPMC RIGHT					***t*_(15)_: 10.91** ***p* <0.0001**	***t*_(15)_:** −**2.69** ***p* = 0.0164**			
M1 LEFT						*t_(15)_: 2.02* *p = 0.0.0612*			*t_(15)_: 0.01* *p = 0.9982*
M1 RIGHT							***t*_(15)_: 4.42** ***p =* 0.0004**	*t*_(15)_*: 0.01* *p = 0.9881*	

*AIP, Anterior IntraParietal; vPMC, ventral PreMotor Cortex; dPMC, dorsal PreMotor Cortex; M1, Primary Motor Cortex. Cells on top of the columns report the “input” region and rows report the “target” region. Values in italic are not significant; values in bold are significant*.

## Discussion

Despite their physical similarity, our two hands tend to play very different roles, with 90% of us showing the right hand dominance when using and interacting with objects, while the left hand has a merely supporting role. Only 10% of the population exhibits the reversed behavioral asymmetry, using the left hand as their dominant one. Thus, it is not surprising that the left-handers have been largely neglected in neuroimaging studies of human motor behavior, with most research focusing only on the right-handed population. To bridge this gap, we investigated the neural underpinnings of precision grasping movements in left-handed participants using a dynamic causal modeling approach (DCM; Friston et al., 2003).

In general, our results confirmed that performing a precision grasping task with either the left or the right hand recruits brain regions belonging to the grasping network, such as the AIP, the vPMC, the dPMC and the M1. We also explored whether and how the intra- and inter-hemispheric causal relationships between “key” cortical nodes of the parieto-frontal grasping network were influenced by the choice of the hand performing the movement in left-handers. For intra-hemispheric connectivity, we focused on the interactions between grasping region, as described by Castiello and Begliomini (2008), that are: AIP, vPMC, dPMC, and M1. For *inter-hemispheric* connectivity we considered two possible scenarios: (i) effective connectivity between homologous areas is affected by precision grasping movements performed with the RNH, given that this hand is supposed to play a “secondary” role with respect to the LDH; (ii) effective connectivity between hemispheres is modulated by the LDH, given the behavioral evidence that even in left-handers the left hand could be less skilled in tasks characterized by high levels of visuomotor processing, such as grasping small objects (Gonzalez et al., 2006, 2007). To test these hypotheses, left-handed participants performed precision grasping movements toward an object with either the right or the left hand.

In terms of the *intra-hemispheric* effective connectivity, we showed that when precision grip movements are performed with the RNH, the connections “AIP-vPMC” and “vPMC-dPMC” within the left hemisphere appear to be significantly modulated. On the other hand, when performing movements with the LDH, the “AIP-vPMC” and “vPMC-dPMC” connections within the right hemisphere were modulated. In addition, the “dPMC-M1” connection appeared to be modulated only within the right hemisphere, when using the LDH. No significant modulation effects were observed in the left hemisphere concerning the use of the RNH for the connection “dPMC-M1.” These results are in line with recent studies showing that effective connectivity between intra-hemispheric nodes of the grasping network is specifically modulated by the choice of the performing hand (Begliomini et al., 2015). However, the fact that only within the right hemisphere (i.e., using the LDH) the final step of the circuit (dPMC-M1) appears to be significantly modulated by the performing hand might suggest that using the LDH requires a stronger “information flow” between these two areas as to accomplish the movement adequately. Overall, the pattern of connectivity observed within hemispheres confirms a series of results obtained in both humans and non-human primates (Rizzolatti and Luppino, 2001; Castiello, 2005; Castiello and Begliomini, 2008), converging on the idea that AIP and both ventral and dorsal regions of the premotor cortex act as “key” areas of the grasping circuit, together with the M1.

When considering *inter-hemispheric* connectivity, results showed that the best fitting models were those hypothesizing a RIGHT → LEFT modulation when the LDH is used. These results speak in favor of a somewhat lower dexterity of the LDH as a modulating factor for inter-hemispheric connectivity between homologous areas. In other words, even if the left hand is supposed to be dominant for left-handers, it might be less skilled to properly accomplish a task requiring high levels of accuracy (i.e., precision grasping). Therefore, additional processing within the ipsilateral (left) hemisphere is required to support the right hemisphere.

Considering the stage at which this bilateral recruitment occurs, connectivity analyses indicated that the AIP and the dPMC are the key nodes for the inter-hemispheric “cross-talk”: connections between the AIPs, as well as the dPMCs, appear to be significantly modulated in both directions. In both humans and non-human primates, the AIP plays a crucial role in “translating” object intrinsic properties into specific grips (Rizzolatti and Luppino, 2001). The present study confirms the bilateral involvement of the AIP in precision grasping tasks, previously observed in right-handers using either the right or the left hand (Tunik et al., 2005; Rice et al., 2006; Davare et al., 2007). For example, Davare et al. (2007) showed that hand shaping, the “core” event of a grasping movement, is impaired only when virtual lesions to both AIP are induced by means of repetitive transcranial magnetic stimulation (rTMS), while no impairment was observed when the AIP lesion was unilateral. The potential existence of a cross-talk between the two AIPs gives further support our present findings. Notably, previous DCM study on right-handers (Begliomini et al., 2015) observed only a LEFT → RIGHT modulation during the execution of precision grasping movements performed with the left non-dominant hand. This result has been explained in terms of additional processing required by the right hemisphere, controlling the less-skilled left hand.

Considering right-handers, the dominance of the left hemisphere when using the right dominant hand in high precision tasks has been testified by many studies (Serrien and Sovijärvi-Spapé, 2015; Króliczak et al., 2016; see Corballis et al., 2012 for a review). The fact that left-handers were characterized by a bi-directional cross-talk when the LDH was used, confirms that the precision grasping task requires additional resources, not only as a result of the complexity of the task, but also because the performing left hand needs additional resources in terms of the visuomotor transformations, even if it is supposed to be the dominant and thus more the “efficient” one.

In a similar vein, the connection between the right and the left dPMC appeared to be modulated in both directions: this observation mirrors the results of a previous study involving right-handers (Begliomini et al., 2015). Other findings in right-handers also indicated that a precision grip performed with the left hand necessitates a contribution of the bilateral dPMC for an appropriate on-line monitoring of the action (Davare et al., 2006; Begliomini et al., 2008, 2015). This evidence provides support to the idea that dPMC plays a crucial role in controlling distal actions, which aligns with neurophysiological evidence showing the presence of neurons selective for the type of prehension in the dorsal premotor cortex of non-human primates (Area F2; Raos et al., 2003).

To summarize, the present study is the first to examine how connections among motor brain areas are affected by hand dominance in left-handers. The results speak in favor of a predisposition of the right hand/left hemisphere for motor tasks requiring high levels of dexterity, such as precision grasping. These results are consistent with those reported by previous behavioral observations (Gonzalez et al., 2006, 2007), suggesting that hemispheric specialization for visuomotor control might be handedness-independent. In this sense, right- and left-handers seem not to differ from each other: the right hemisphere involved in supporting the ongoing action recruits resources also from the left hemisphere to accomplish the action successfully. More precisely, performing a precision grasping task with the left hand highlights boosted inter-hemispheric connections between homologous areas (AIP and dPMC), suggesting the need of additional resources in terms of both visuomotor processing (AIP) and on-line monitoring (dPMC), both required to accomplish the action in an efficient manner. Additional studies on larger cohorts of left-handers (Mazoyer et al., 2016), and including populations characterized by different degrees of right- and left-handedness would be beneficial for a fine-grained exploration of the role of handedness in motor control.

In conclusion, the present study further validates neurophysiological and neuroimaging data on the cortical control of grasping in humans, adding novel insights on the intra- and inter-hemispheric interplay underlying grasping actions. Our results also contribute to fill the gap of knowledge on motor control in left-handers, shedding new light on the sophisticated interplay between handedness and motor control.

## Author contributions

Experiment Conception: CB, LS, and UC; Experiment Data Collection: CB and LS; Experiment data analysis: CB, MD, and SB; Manuscript preparation: CB, LS, MD, SB and UC.

### Conflict of interest statement

The authors declare that the research was conducted in the absence of any commercial or financial relationships that could be construed as a potential conflict of interest.
